# The TRPM6/EGF Pathway Is Downregulated in a Rat Model of Cisplatin Nephrotoxicity

**DOI:** 10.1371/journal.pone.0057016

**Published:** 2013-02-15

**Authors:** Kristien J. Ledeganck, Gaëlle A. Boulet, Johannes J. Bogers, Gert A. Verpooten, Benedicte Y. De Winter

**Affiliations:** 1 Laboratory of Experimental Medicine and Pediatrics, University of Antwerp, Antwerp, Belgium; 2 Applied Molecular Biology Research Group, University of Antwerp, Antwerp, Belgium; 3 Department of Nephrology-Hypertension, Antwerp University Hospital, Edegem, Belgium; Johns Hopkins University, United States of America

## Abstract

Cisplatin-induced hypomagnesemia is described in humans and rats, but the underlying mechanisms are still unclear. Recent studies have shown that epidermal growth factor (EGF) stimulates Mg^2+^ re-absorption in the distal convoluted tubule via the Mg^2+^ channel TRPM6. This study investigates the role of TRPM Mg^2+^ channels, claudines, and EGF in the Mg^2+^ homeostasis in a rat model of cisplatin-induced nephrotoxicity. Wistar rats were given 2.5 mg/kg cisplatin per week for 3 weeks and were euthanized 4 or 9 weeks after the first administration. The cisplatin treatment significantly increased the fractional excretion of Mg^2+^. Real-time RT-PCR and/or Western blots were performed to assess the renal expression TRPM6, TRPM7, claudin-16, claudin-19, EGF, EGF receptor (EGFR) and EGFR-pathway components. The renal mRNA expression of TRPM6 and EGF showed a significant decrease after cisplatin treatment, while the TRPM7, claudin-16 and EGFR expressions remained stable. The claudin-19 mRNA expression was significantly upregulated after cisplatin treatment. Western blotting confirmed the mRNA expression data for the claudins, but an showed upregulation of EGFR only at week 9. The role of the EGFR pathway, involving Pi3-AKT-Rac1, in cisplatin-induced nephropathy, could not be substantiated in further detail. This study shows that cisplatin treatment results in EGF and TRPM6 downregulation in the rat kidney, causing renal Mg^2+^ loss. Our results are in line with the hypothesis that EGF influences the renal expression or activation of TRPM6 and plays a significant role in Mg^2+^ loss in medication-induced nephropathy.

## Introduction

About 43% of the patients treated with cisplatin develop hypomagnesemia due to renal magnesium (Mg^2^^+^) loss [1]. Moreover, it was shown that acute MgCl_2_ infusions lead to a significantly higher urinary Mg^2^^+^ excretion in cisplatin treated rats and humans [2], [3]. In rats, the Mg^2^^+^ depletion enhances cisplatin-induced nephrotoxicity, significantly increasing plasma creatinine and plasma urea [4]. However, the mechanisms underlying these observations have not yet been described.

The major site of passive Mg^2^^+^ re-absorption is the thick ascending limb (TAL) where 70% of the Mg^2^^+^ is reabsorbed. The tight junction proteins claudin-16 (also known as paracellin-1) and claudin-19 are the key players in this paracellular transport [5], [6].

Recently, two other ion channels with an important role in the Mg^2^^+^ homeostasis were identified, TRPM6 and TRPM7. They belong to the transient receptor potential subfamily Melastatin (TRPM). TRPM6 is predominantly expressed in absorbing epithelia. In the kidney, TRPM6 is present in the distal convoluted tubule (DCT), known as the main site of active transcellular Mg^2^^+^ re-absorption along the nephron. TRPM7 is ubiquitously expressed and implicated in cellular Mg^2^^+^ homeostasis, cell death, and cell cycle regulation [7], [8].

Our research group and others reported that hypomagnesemia in calcineurin inhibitor (CNI)-induced nephropathy is related to the downregulation of epidermal growth factor (EGF) and TRPM6 [9], [10], [11]. Recently, it was shown that EGF stimulates Mg^2^^+^ re-absorption in the DCT via its receptor on the basolateral membrane and via activation of TRPM6 in the apical membrane. The EGF-mediated stimulation of TRPM6 occurs through signaling via SRC kinases and Rac1 *in vitro*, thereby redistributing endomembrane TRPM6 to the plasma membrane [12], [13]. This pathway has not been studied thoroughly in cisplatin-induced nephropathy although it was shown that cisplatin administration results in a decreased renal expression of EGF and a decreased urinary EGF excretion in the rat [14].

This study aimed to unravel the molecular mechanisms of cisplatin-induced hypomagnesemia. The expression profiles of several proteins involved in the Mg^2+^ re-absorption, i.e. TRPM6, TRPM7, claudin-16, claudin-19 and EGF, were analyzed as well as the interaction of TRPM6 with EGF. Moreover, the role of the EGF receptor (EGFR) and its signaling pathway was studied through the expression analysis of several signaling proteins.

## Materials and Methods

### Animal model

Twenty-four male Wistar rats (Charles River, Brussels, Belgium), weighing between 180 and 200 g, were housed two to four per cage, at 22 ± 2°C with a 12h dark-light cycle. They had free access to water and were fed a normal diet (0,30% Mg, Carfil Labofood, Oud-Turnhout, Belgium).

The ethical approval for the animal studies was obtained from the Medical Ethical Committee on Animal Experimentation of the University of Antwerp, Belgium (file 2009/17).

### Experimental set-up

Rats were divided into two groups (each containing twelve animals) and treated as follows.

Group 1 (control rats): the rats received a weekly intraperitoneal injection of saline in a volume equal to the cisplatin-treated group during 3 weeks.

Group 2 (cisplatin-treated rats): the rats received a weekly intraperitoneal injection of cisplatin (2.5 mg/kg/w, Cisplatin Hospira 100 mg/100 ml ONCO-TAIN^®^, Hospira, Warwickshire, UK) during three weeks. This dose of cisplatin and the three-week time interval between the first administration and the euthanasia result in minimal tubular damage and allows a focus on the functional effects in the rat model. A previous study showed that this cisplatin treatment scheme causes hypomagnesemia in the rat [2].

This experiment was performed twice to obtain data 4 or 9 weeks after the first cisplatin administration.

### Sample collection

One week before the first cisplatin administration, the rats were caged individually in a metabolic cage to obtain a 5-hour urine collection. At the same time, 0.6 ml blood was collected from the tail vein. Twenty-eight days after the first cisplatin/saline administration, the rats were caged individually to obtain a second 5-hour urine collection. Afterwards, six rats of each group were euthanized by an overdose of pentobarbital (100-150 mg/kg) intraperitoneally. Blood samples were taken from the vena cava inferior at the time of sacrifice. The kidneys were quickly prelevated and decapsulated. The right kidney was snap-frozen in liquid nitrogen and stored at -80°C until the RNA extraction was performed. The left kidney was cut into 2 mm thick transverse slices and processed for further histological analysis using different fixation procedures (described below). To evaluate the long term effect of cisplatin, the six remaining rats of each group were sacrificed 63 days after the first dose of cisplatin.

### Serum and urine creatinine, Mg^2^^+^, Na^+^ and K^+^ levels

Serum and urine creatinine, Mg^2+^, Na^+^ and K^+^ levels were measured using an indirect potentiometric method with a dimension Vista 1500 System (Siemens Healthcare Diagnostics, Deerfield, USA).

The fractional excretion (FE) of Mg^2+^ was calculated as: FE_Mg_  =  100×(U_Mg_×P_Cr_)/[(0.7×P_Mg_) ×U_Cr_], with U_Mg_ urinary excretion of Mg^2^^+^ (mg/dl), P_Cr_ plasma creatinine (mg/dl), P_Mg_ plasma Mg^2^^+^ (mg/dl) and U_Cr_ urinary excretion of creatinine.

The FE of Na^+^ was calculated as: FE_Na_  =  100× (U_Na_×P_Cr_)/(P_Na_×U_Cr_). With U_Na_ urinary excretion of Na^+^ (mmol/l), P_Cr_ plasma creatinine (mg/dl), P_Na_ plasma Na^+^ (mmol/l) and U_Cr_ urinary excretion of creatinine.

### Morphological assessment of cisplatin-induced lesions

For light microscopy, the rat kidney slices were fixed in formol during 24h, embedded in paraffin, cut in 5 ∶m-sections and stained with periodic acid-Schiff reagent or Sirius red collagen stain. Sirius red-stained sections were scanned with an Olympus BX61 Motorized Research Microscope (Olympus Corporation, Tokio, Japan) equipped with AnalySIS pro 5.0 software (Olympus). After the processing of the image, the total amount of collagen was measured and expressed as the percentage of the total cortex.

### Quantification of inflammatory cells

The formol-fixed, paraffin-embedded renal tissue was stained with the ED1 monoclonal antibody (Acris Antibodies, Hiddenhausen, Germany), directed to an antigen of tissue macrophages and peripheral blood granulocytes. Infiltration was quantified by counting immunoreactive cells in 28 randomly chosen areas (magnification×200) of the cortex [15]. An observer, blinded for the experimental groups, examined 30 glomeruli per renal section. Inflammatory cells contained within large blood vessels or surrounded by erythrocytes were excluded from all counts. The results were expressed as absolute numbers of immunoreactive cells per mm^2^ or per glomerular cross section.

### mRNA isolation and cDNA synthesis

Total RNA was extracted from the kidneys using a column-based technique (RNeasy Minikit, Qiagen, KJ Venlo, Netherlands). Purified total RNAs were treated with DNase to obtain DNA-free RNA (Turbo DNase free, Ambion, Applied Biosystems, Lennik, Belgium). cDNA was synthesized using the Transcriptor First Strand cDNA Synthesis Kit (AMV) (Roche Applied Science, Indianapolis, IN, USA).

### Real-time PCR

To examine the mRNA expression of TRPM6, EGF, EGFR, Pi3, Akt and Rac1, real-time PCRs were performed using the LightCycler Taqman Master (Roche, Vilvoorde, Belgium). TGF-β, PAI-1, TRPM7, claudin-16 and claudin-19 mRNA expressions were examined using the LightCycler FastStart DNA Master plus SYBR green I (Roche, Vilvoorde, Belgium). The PCRs were performed as previously described [9]. All genes were normalized against the housekeeping gene glyceraldehyde 3-phosphate dehydrogenase (GAPDH), which has been described as a stable reference gene for renal tissue in this experimental set-up [16] and expressed as the normalized ratio (NR). The renal sodium chloride channel (NCC) mRNA expression was determined to evaluate the distal tubule damage.

### Western blotting

Kidney tissue was mechanically homogenized, using a Polytron Homogenizer, in lysis buffer (containing a protease inhibitor cocktail (cOmplete Mini, Roche Applied Science) and a phosphatase inhibitor cocktail (PhosSTOP, Roche Applied Science) in 20 mM Tris HCl, 137 mM NaCl, 10% glycerol, 1% nonidat-40 and 2 mM EDTA) to collect whole tissue lysates for immunoblotting. Sixty-five μg protein was separated electrophoretically on a 4-12% NuPage gel (Invitrogen, Gent, Belgium) and electrotransferred onto PVDF membranes. The membranes were blocked with 5% bovine serum albumin in Tris buffered saline plus 0,1% Tween for 1 h at room temperature. Specific primary antibodies were applied overnight: anti-TRPM6 (Abgent, San Diego, USA), anti-TRPM7 (Abcam, Cambridge, USA), anti-claudin-16 (Santa Cruz Biotechnologies, California, USA), anti-Claudin-19 (Santa Cruz Biotechnologies), anti-EGFR (Cell Signaling Technology, Danvers, USA), anti-Pi3 Kinase (Millipore, Billerica, USA), anti-Akt (Cell Signaling Technology) and anti-Rac1 (Abcam). Blots were visualized using the SuperSignal West Pico substrate (Pierce-Thermo Scientific, Rockford, USA). Densitometric analyses were performed using the Lumianalyst® 3.1 software (Roche Molecular Biochemicals). The protein intensities were normalized against a housekeeping protein in the same lane (Cox IV, Cell Signaling Technology).

### Statistics

The results are presented as a mean ± SD. The differences between the cisplatin-treated groups and the controls at a specific time point (4 or 9 weeks) were analyzed using generalized linear models, which included ‘group’ and ‘time after treatment’ as predictors for each different dependent variable. The correlation between TRPM6 mRNA and EGF mRNA was measured using a Pearson correlation coefficient. Statistical analysis was performed using SPSS (version 20.0) for Windows. P-values less than 0.05 were considered statistically significant.

## Results

### Functional parameters (table 1)

**Table 1:** Urine and serum analyses and body weight.

	Controls w4	Cisplatin 4w	Controls 9w	Cisplatin 9w
Body weight (g)	375 ± 37	305 ± 17^ a^	406 ± 26	358 ± 37^ b^
Serum Creatinine (mg/dl)	0.30 ± 0.07	0.42 ± 0.04 ^a^	0.27 ± 0.03	0.54 ± 0.13^ b^
Serum Mg^2+^ (mg/dl)	2.60 ± 0.40	2.71 ± 0.24	2.55 ± 0.34	2.90 ± 0.32
Serum Na^+^ (meq/l)	143.13 ± 3.21	147.37 ± 5.57	146.00 ± 6.47	146.50 ± 4.35
Serum K^+^ (meq/l)	5.66 ± 1.80	5.51 ± 0.61	5.51 ± 0.33	5.27 ± 0.90
FE Mg^2+^ (%)	9.23 ± 4.52	32.56 ± 21.50^ a,b^	9.06 ± 3.08	20.15 ± 8.60
FE Na^+^ (%)	0.19 ± 0.11	0.18 ± 0.15	0.11 ± 0.06	0.24 ± 0.20

Data are presented in 4 groups: control animals receiving vehicle only, cisplatin-treated animals (2.5 mg/kg/w during 3 weeks) at 4 and 9 weeks after treatment with vehicle or cisplatin. Data are presented as means ± SD. Statistics were performed using generalized linear models. ^a^p<0.05 versus controls at 4 weeks, ^b^p<0.05 versus controls at 9 weeks.

No rats died during the experiment. All experimental groups showed similar body weights and laboratory values (serum creatinine, creatinine clearance, serum Mg^2^^+^, FE Mg^2+^, serum Na^+^, serum K^+^ and FE Na^+^) at the start of the study (data not shown). At the time of sacrifice, the mean body weight in the control groups was significantly higher than in the cisplatin-treated groups (p<0.05). The serum creatinine values were significantly higher in the cisplatin-treated group after 4 weeks of treatment (p = 0.004) and after 9 weeks (p = 0.003) *versus* controls. The serum Mg^2^^+^ concentration did not differ significantly between the cisplatin-treated groups and the controls. The FE Mg^2+^ was significantly higher in the cisplatin-treated group after 4 weeks (p<0.001) and was increased after 9 weeks *versus* the controls, although not significantly (p =  0.082). The serum Na^+^ , serum K^+^ and the FE Na^+^ were comparable in all groups.

### Morphological assessment of cisplatin-induced lesions (Figure 1)

**Figure 1:**
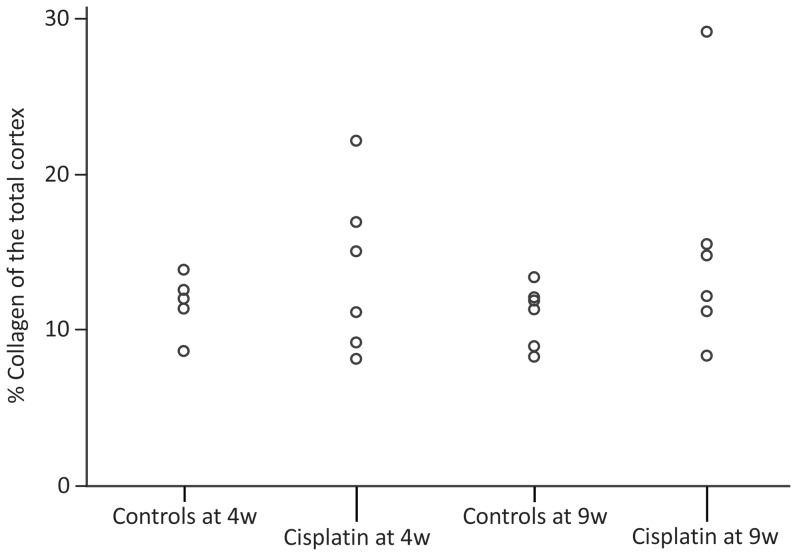
% Collagen of the total cortex. Effect of cisplatin on the quantity of collagen in the renal cortex. Sirius red-stained sections of all rats were scanned, the total amount of collagen was measured and expressed as percentage of the total cortex. Data are presented per group per rat. Statistics were performed using generalized linear models.

In each cisplatin-treated group, one rat showed signs of increased interstitial fibrosis on PAS-stained sections. However, the experimental groups showed no difference in interstitial fibrosis: the mean score of interstitial fibrosis was 13.87 ± 5.25% in cisplatin-treated rats at 4 weeks *versus* 11.58 ± 1.44% in the control group (p = 0.343). At 9 weeks, the mean score of interstitial fibrosis was 15.10 ± 7.52% in the cisplatin-treated rats *versus* 10.74 ± 1.81% in the control group (p = 0.221).

### Quantification of inflammatory cells (Figure 2)

**Figure 2:**
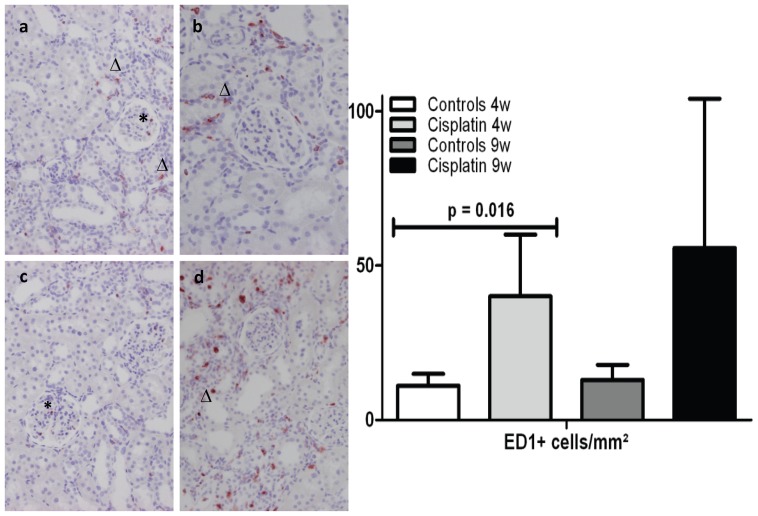
Interstitial and glomerular ED1 infiltration. Left part: ED1 positive cell in a glomerulus (*) and in the interstitium (▵) of a) a control rat at 4 weeks; b) a cisplatin-treated rat at 4 weeks; c) a control rat at 9 weeks and d) a cisplatin-treated rat at 9 weeks. Right part: Effect of cisplatin on the infiltration of ED1-positive cells. Results are expressed as the mean ± SD of the absolute numbers of immunoreactive cells per mm^2^, each group containing n = 6. Statistics were performed using generalized linear models.

The control rats showed very few ED1-positive cells in the cortex. A significant infiltration of ED1-positive cells was seen in the cortex of the rats treated with cisplatin at 4 weeks (p = 0.016). This was not longer the case at week 9 (p = 0.084).

### Real-time RT-PCR

**Figure 3:**
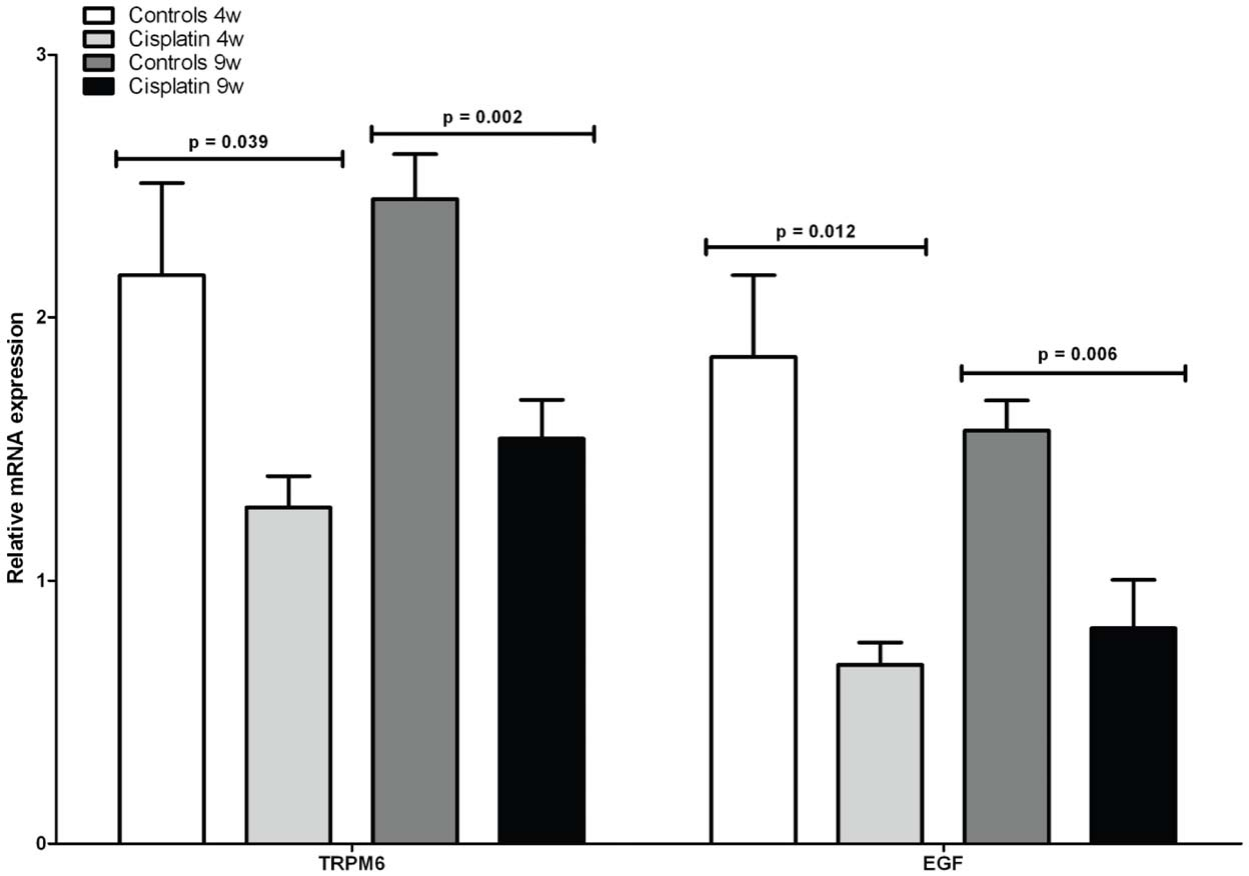
mRNA expression levels of TRPM6 and EGF. Effect of cisplatin on renal mRNA expression levels of Mg^2+^ transport protein TRPM6 and EGF in the rat kidney. Data are presented as means ± SD, each group containing n = 6. Statistics were performed using generalized linear models.

The renal mRNA expression of TRPM6 (Figure 3) significantly decreased in the cisplatin-treated group at 4 weeks (NR: 1.28 ± 0.29) *versus* controls (2.16 ± 0.86, p = 0.002) and at 9 weeks (NR: 1.54 ± 0.36) *versus* controls (NR: 2.45 ± 0.42, p = 0.001).

The renal mRNA expression of EGF (Figure 3) significantly decreased in the cisplatin-treated group at 4 weeks (NR: 0.68 ± 0.21) *versus* controls (1.85 ± 0.76, p<0.001) and at 9 weeks (NR: 0.82 ± 0.45) *versus* controls (NR: 1.57 ± 0.28, p = 0.003).

**Figure 4:**
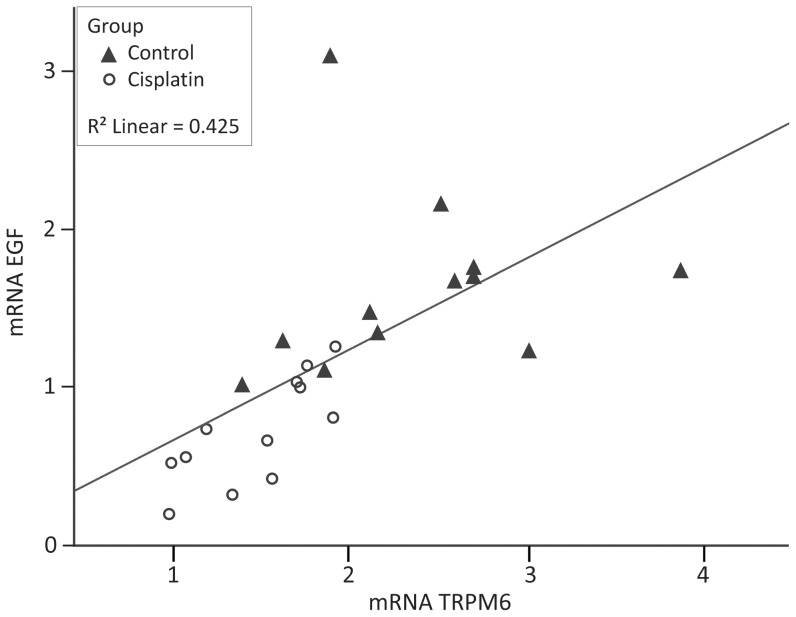
correlation between mRNA expression levels of TRPM6 and EGF. Data are presented per rat per group (▴controls, ○ cisplatin-treated rats). TRPM6 mRNA correlates with EGF mRNA expression level. Statistics were performed using a Pearson correlation coefficient. EGF, epidermal growth factor.

The renal mRNA expression of EGF correlated significantly with the TRPM6 mRNA expression (p = 0.001). As shown in figure 4, a low renal mRNA expression of EGF is associated with a low renal mRNA expression of TRPM6.

**Figure 5:**
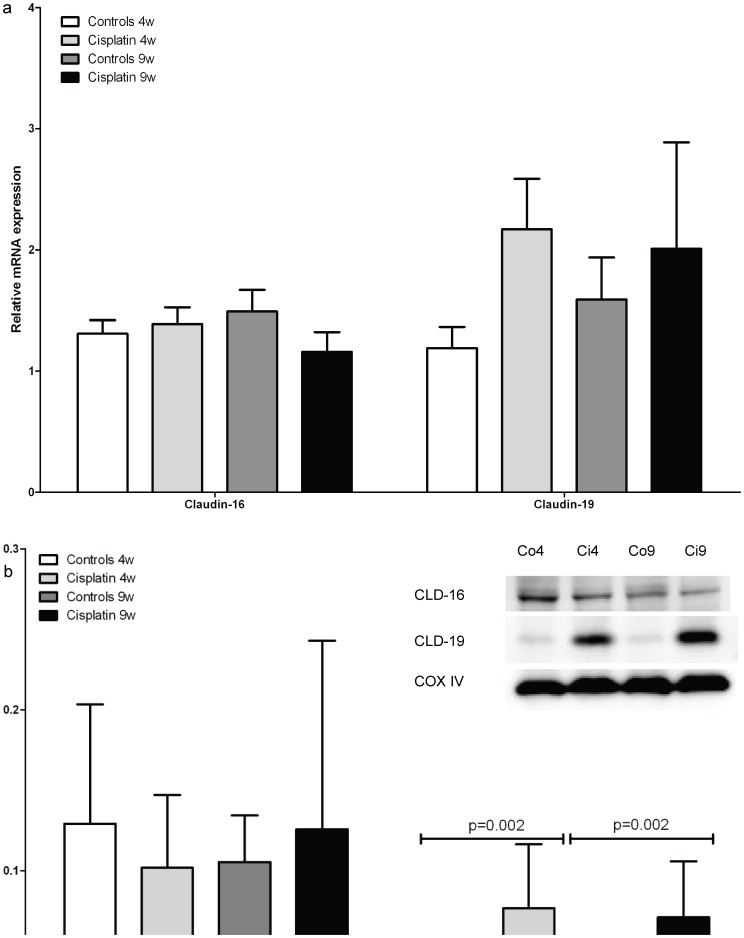
mRNA and protein expression levels of claudin-16 and claudin-19. a) Effect of cisplatin on renal mRNA expression levels of tight junction proteins claudin-16 and claudin-19. b) Expression of claudin-16 and claudin-19 protein in kidney tissue of rats was examined with Western blot analyses using claudin-16 and claudin-19 antibody (described in materials and methods). Data are presented as means ± SD, each group containing n = 6. Statistics were performed using generalized linear models. Immunoblots reveal a band at 22kD (claudin-19) and 34kD (Claudin-16). CLD16, claudin-16; CLD19, claudin-19; Co4, control rats at 4 weeks, Ci4, cisplatin-treated rats at 4 weeks, Co9, control rats at 9 weeks, Ci9, cisplatin-treated rats at 9 weeks.

The groups showed no significant differences in renal mRNA expression of TRPM7 (data not shown) and claudin-16, whereas claudin-19 showed a tendency to increase although no significance was reached (p = 0.144 at 4 weeks and p = 0.530 at 9 weeks, Figure 5). The renal mRNA expression of EGFR did not differ between the cisplatin-treated and the controls (table 2).

**Table 2:** mRNA and protein expression levels of EGFR, Pi3, Akt and Rac1.

	Controls 4w		Cisplatin 4w		Controls 9w		Cisplatin 9w	
	mRNA (NR)	Protein (%)	mRNA (NR)	Protein (%)	mRNA (NR)	Protein (%)	mRNA (NR)	Protein (%)
EGFR	1.67 ± 0.44	100.00 ± 26.96	1.91 ± 0.61	442.54 ± 160.09	1.96 ± 0.79	100.00 ± 6.82	2.29 ± 1.28	397.56 ± 167.29^a,b^
Pi3	10.34 ± 5.15	100.00 ± 37.77	17.32 ± 7.05	316.42 ± 86.44^a^	12.55 ± 6.57	100.00 ± 29.76	57.84 ± 41.29^a,b^	121.01 ± 36.51
Akt	2.41 ± 1.01	100.00 ± 25.98	1.72 ± 1.12	326.75 ± 37.63^a,b^	2.03 ± 1.95	100.00 ± 10.27	7.29 ± 5.35^a,b^	206.63 ± 55.69^a,b^
Rac1	5.22 ± 2.15	100.00 ± 44.14	5.22 ± 1.93	68.29 ± 11.11	4.65 ± 2.40	100.00 ± 12.94	11.30 ± 9.58^a,b^	110.68 ± 24.91

Data are presented in 4 groups: control animals receiving vehicle only, cisplatin-treated animals (2.5 mg/kg/w during 3 weeks) at 4 and 9 weeks after treatment with vehicle or cisplatin. Protein expression levels are presented as % ± SD compared to the control group at 4 weeks and at 9 weeks. mRNA expression levels are presented as mean normalized ratio (NR) ± SD. Statistics were performed using generalized linear models. ^a^p<0.05 versus controls at 4 weeks, ^b^p<0.05 versus controls at 9 weeks.

Pi3, Akt and Rac1 mRNA expression showed no differences at week 4 while their expression was significantly upregulated in the cisplatin-treated rats *versus* controls at week 9 (p<0.001, p<0.001 and p = 0.028 respectively, table 2).

PAI-1 was upregulated in both cisplatin-treated groups, although not sigificantly. The NR was 8.48 ± 10.48 in the cisplatin-treated group after 4 weeks *versus* 1.49 ± 0.33 in the control group (p = 0.201). At 9 weeks, the NR was 12.35 ± 17.89 in the cisplatin-treated group versus 1.70 ± 0.84 in the control group (p = 0.052).

TGF-β was significantly upregulated in the cisplatin-treated group (NR: 3.22 ± 1.54) at 4 weeks *versus* controls (1.19 ± 0.28, p = 0.015) and at 9 weeks (NR cisplatin-treated rats: 3.21 ± 2.68 and NR controls: 1.13 ± 0.50, p = 0.012).

Renal NCC mRNA expression levels did not differ between the cisplatin-treated groups and the controls at 4 weeks (NR controls: 1.24 ± 0.42, NR cisplatin–treated rats: 1.21 ± 0.69, p = 0.890). At 9 weeks, the NCC mRNA expression levels were downregulated in the cisplatin-treated rats (NR controls: 1.80 ± 0.58, NR cisplatin-treated rats: 0.66 ± 0.23, p<0.001).

### Western blotting

Western blot analysis for TRPM6 and TRPM7 could not be executed due to the unavailability of the appropriate specific primary anti-rat antibodies.

There was no difference in the claudin-16 protein expression level between the control and cisplatin-treated groups (p = 0.476 at 4 weeks and p = 0.627 at 9 weeks, Figure 5). Claudin-19 was significantly upregulated in both cisplatin-treated groups (817 ± 295 %, p<0.001 at 4 weeks and 665 ± 273 %, p<0.001 at 9 weeks, Figure 5).

The EGFR protein expression did not differ at 4 weeks, but was significantly increased at 9 weeks (p = 0.013, table 2). Pi3 was significantly upregulated at 4 weeks *versus* controls (p = 0.006), but not at 9 weeks (p = 0.661, table 2). Akt1 was significantly upregulated in the cisplatin groups at 4 weeks (p<0.001) and at 9 weeks (p = 0.017, table 2). Rac1 protein expression was stable in all groups at 4 weeks and at 9 weeks (table 2).

## Discussion

This *in vivo* study shows that cisplatin downregulates the EGF and TRPM6 mRNA levels while the FE Mg^2+^ increases and therefore suggests that cisplatin treatment results in a diminished renal Mg^2^^+^ re-absorption via the downregulation of the Mg^2+^ channel TRPM6.

The rat model showed an increased serum creatinine and an increased FE Mg^2+^ after 4 weeks of treatment, conform to what has been seen in other studies [2], [17]. Our rat model showed the pathogenic characteristics of cisplatin nephrotoxicity: the pro-fibrotic factor TGF-β was upregulated as previously shown [18]. The same was true for PAI-1, which also plays a role in the progression to fibrosis [19]. In addition to this pro-fibrotic environment, a pro-inflammatory environment with an infiltration of ED1-positive cells in the interstitium was seen at week 4. However, the measurement of the total amount of collagen showed no increase in interstitial fibrosis after cisplatin treatment. Therefore, the kidney damage remained limited to a pro-inflammatory pro-fibrotic condition.

This study focused on the role of TRPM6 and EGF in cisplatin-induced renal Mg^2+^ loss. A simultaneous downregulation of the renal TRPM6 and EGF mRNA expression was found together with an increased FE Mg^2+^. The renal EGF expression correlated well with the renal TRPM6 mRNA expression. Cisplatin administration is known to result in a decreased renal EGF expression and urinary EGF excretion in the rat [14]. However, the link between renal EGF expression and renal Mg^2+^ loss has not yet been established in this cisplatin rat model. Previously, our group proved the relation between CNI-induced renal Mg^2+^ loss and TRPM6 downregulation in a cyclosporine (CsA) ratmodel [9]. In the CsA rat model, we found the simultaneous downregulation of the renal EGF mRNA expression. We hypothesize that the mechanism leading to hypomagnesemia is similar in rats treated with cisplatin and rats treated with CNI and is related to a decreased expression of the renal Mg^2^^+^ channel TRPM6.

The reduced expression of EGF in rats treated with cisplatin is in accordance with a mechanism, which was previously revealed *in vitro.* EGF stimulates the Mg^2^^+^ re-absorption in the DCT via its receptor on the basolateral membrane and via activation of TRPM6 in the apical membrane *in vitro* [12], [13]. However, the results from the present study together with the results of our previous study of CsA-induced nephrotoxicity suggest that an additional EGF-induced mechanism is involved in TRPM6 regulation. Both studies show simultaneous TRPM6 and EGF mRNA downregulation, which might indicate that EGF also influences the TRPM6 mRNA synthesis [9]. Also the finding of an upregulated TRPM6 mRNA expression in rats treated with EGF in our previous study further supports this hypothesis [9]. To further elucidate the relationship between EGF and TRPM6 *in vivo*, the effect of cisplatin on the EGFR pathway was studied. Based on the activation mechanism of TRPM6 via EGF, as established *in vitro*, Pi3, Akt and Rac1 expression profiles were evaluated. Activation of these EGFR pathway members leads to TRPM6 activation and redistribution [13]. However, the present study could not establish a straightforward association between the cisplatin-induced EGF downregulation and the down-stream activation of the EGFR pathway members. This could be related to the fact that the investigated intermediates are activated in many other pathways involved in kidney injury such as the insulin-dependent Pi3/Akt activation, the platelet-derived growth factor-induced Pi3/Akt pathway and the TGF-β1-increased collagen1 expression through the Pi3-PDK1-AKT pathway [20], [21], [22]. All these pathways are potentially upregulated in the cisplatin-induced nephrotoxicity rat model. Moreover, in the present study, the total cortex protein and mRNA levels were analyzed, which might be a too crude extract to detect specific DCT related changes in the Pi3/Akt pathway.

However, there is evidence for the EGFR pathway-mediated activation of TRPM6 *in vivo.* Dimke *et al.* studied the effect of an EGFR inhibitor on renal Mg^2+^ handling. Erlotinib-injected mice failed to reduce the FE Mg^2+^ in response to a decreased serum Mg^2+^ concentration. TRPM6 mRNA was downregulated but not the TRPM6 protein level, indicating that the hypomagnesemia is due to the inactivation of TRPM6 at protein level [23]. Furthermore, several clinical studies reported hypomagnesemia due to renal Mg^2+^ wasting after treatment with EGFR-inhibiting antibodies [24], [25], [26].

The mRNA expression levels of the EGFR remained unchanged. EGFR protein expression remained unchanged after 4 weeks but increased after 9 weeks. This up-regulation is probably a reaction in order to repair cisplatin-induced tubular damage [27].

TRPM7 is very homologue to TRPM6 (≈50% homology) and also responsible for the cellular Mg^2^^+^ homeostasis [28], [29]. TRPM6 specifically interacts with TRPM7 to form a functional ion channel complex at the cell surface of human embryonic kidney 293 cells [30], [31]. In our study, the cisplatin-treated groups showed no TRPM7 downregulation. At this point, it is unclear whether TRPM6 and TRPM7 are both expressed in the DCT of rat kidneys, which is the case for mice and humans [32], [33]. Immunocytochemical stainings for both channels were unsuccesful due to the unavailability of appropriate primary anti-rat antibodies.

Since NCC is only expressed in de DCT, renal NCC mRNA expression and FE Na^+^ were measured to evaluate DCT damage [34]. These parameters did not differ between the control group and the cisplatin-treated group after 4 weeks, suggesting that the TRPM6 downregulation is not due to damaged DCTs. This is in contrast to a recent report which describes the downregulation of 3 DCT markers (TRPM6, NCC and Parvalbumine) after cisplatin-treatment in mice and concluded that cisplatin affects the entire DCT, leading to renal Mg^2+^, K^+^, Na^+^ and Ca^2+^ wasting [35]. The main difference with our rat model is that we induced low grade functional nephrotoxicity, leading to a selective down regulation of TRPM6, while Van Angelen *et al.* induced in mice tubular necrosis using a higher dose of cisplatin and less time between the consecutive cisplatin administrations, leading to acute DCT necrosis with downregulation of all DCT markers. Our data indicate a time-related negative effect of cisplatin on the NCC, with a decreased NCC expression level at 9 weeks. However, the FE Na^+^ was stable which reflects the maintenance of the sodium re-absorption by compensatory regulatory mechanisms. Moreover, at 9 weeks, the TRPM6 mRNA expression and the FE Mg^2+^ partially recovered compared to the cisplatin-treated group at 4 weeks.

In our rat model, the cisplatin-induced Mg^2^^+^ loss is DCT-specific. The mRNA expression of the tight junction proteins claudin-16 (also known as paracellin-1) and claudin-19, which are the key players of the paracellular Mg^2^^+^ transport of the TAL, did not differ between the cisplatin-treated rats and the control rats [5], [6]. Claudin-19 protein expression increased at 4 and at 9 weeks, which suggests the activation of a compensatory mechanism for the TRPM6 downregulation.

In conclusion, this study shows that cisplatin treatment results in EGF and TRPM6 downregulation in the rat kidney, causing renal Mg^2+^ loss. Our results are in line with the hypothesis that EGF influences the renal expression or activation of TRPM6 and plays a significant role in Mg^2+^ loss in medication-induced nephropathy.
